# Luminous Upconverted Nanoparticles as High-Sensitivity Optical Probes for Visualizing Nano- and Microplastics in *Caenorhabditis elegans*

**DOI:** 10.3390/s25113306

**Published:** 2025-05-24

**Authors:** Bushra Maryam, Yi Wang, Xiaoran Li, Muhammad Asim, Hamna Qayyum, Pingping Zhang, Xianhua Liu

**Affiliations:** 1 School of Environmental Sciences and Engineering, Tianjin University, Tianjin 300072, China; maryam_bushra@tju.edu.cn (B.M.); wangyi2023@tju.edu.cn (Y.W.); lxr_11281209@tju.edu.cn (X.L.); 2 Shandong Lead Chemicals Co., Ltd., Linyi 276100, China; asim.muhammad100@yahoo.com; 3 National Synchrotron Radiation Laboratory, University of Science and Technology of China, Hefei 230029, China; hamna141987@mail.ustc.edu.cn; 4 College of Food Sciences and Engineering, Tianjin Agricultural University, Tianjin 300384, China

**Keywords:** upconverted nanoparticles, polystyrene, visualization, nanoplastics, microplastics, *Caenorhabditis elegans*

## Abstract

With the increasing prevalence of plastic pollution, understanding its impact on soil nematodes is crucial for environmental sustainability and food security. Traditional fluorescence-based probes have the limitations of high background noise and interference from autofluorescence. In this study, the luminous upconverted NaYF4:Yb^3+^/Er^3+^ nanoparticles acted as high-sensitivity probes for real-time visualization of ingestion and biodistribution of polystyrene microplastics (PS-MPs) and nanoplastics (PS-NPs) in *Caenorhabditis elegans*. The novel probes enabled efficient near-infrared-to-visible light conversion. This approach improved the precision of nano- and microplastic detection in biological tissues. Microscopic imaging revealed that the probes effectively distinguished size-dependent plastic distribution patterns, with microplastics remaining in the digestive tract, whereas nanoparticles penetrated intestinal walls and entered systemic circulation. Quantitative fluorescence analysis confirmed that PS-NPs exhibited higher bioavailability and deeper tissue penetration, providing crucial insights into plastic behavior at the organismal level. The different toxicities of PS-NPs and PS-MPs were further confirmed by measurement of the locomotor impairments and the physiological disruptions. These findings emphasize the broader applications of upconverted nanoparticles as advanced bio-imaging probes, offering a sensitive and non-invasive tool for tracking pollutant interactions in environmental and biological systems.

## 1. Introduction

Plastics are widely used owing to their versatility [1]; however, most of them eventually enter the environment, where they degrade into long-lasting microplastics (MPs, <5 mm) and nanoplastics (NPs, <1 μm) [2,3,4]. Contamination of soil by nano- and microplastics (NMPs) is an emerging environmental concern. Once introduced into the soil, NMPs can provide amplified environmental and biological hazards due to their tiny size and persistence [5]. They can additionally impact soil structure, reduce water retention, and alter microbial populations, potentially threatening soil health and ecosystem processes [6]. Among various plastics, polystyrene is one of the most widely used polymers, commonly employed in food packaging and as a soil conditioner to improve structure and moisture retention [7]. NMPs have been identified in organic fertilizers, sewage sludge, and wastewater, which are significant sources in terrestrial ecosystems [8,9,10]. The extensive prevalence of these NMPs in agriculture underscores the pressing necessity for sophisticated methods to precisely monitor them and analyze their biological effects [11,12].

NMPs may be consumed by soil organisms, including worms, leading to physical obstructions and chemical toxicity. Furthermore, NMPs may absorb and convey destructive contaminants, including heavy metals and pesticides, hence intensifying their adverse impacts on soil worms [13,14]. Moreover, the presence of NMPs might modify the structure and nutritional composition of soil, hence altering the habitat and food supplies for soil worms [15,16]. This disturbance may result in a reduction in worm populations, thus impacting soil fertility and health, as soil worms are essential for decomposing organic waste and facilitating nutrient cycling [17,18]. *Caenorhabditis elegans* (*C. elegans*) is a free-living worm that has typical characteristics of a model-animal, such as a short life cycle, transparent body, miniscule size, and ease of cultivation. Consequently, it serves as an exemplary model organism for toxicity assessment [19]. To study the interaction between NMPs and soil micro-organisms, it is crucial to track and visualize NMPs within these organisms. Numerous studies have employed fluorescent particles, specifically surface-functionalized polystyrene nanoparticles, as models for PS-NMPs. This approach encounters obstacles, including low detection sensitivity and interference from background fluorescence, which frequently restricts conventional fluorescence-based imaging [20].

Upconversion (UC) nanoparticles, which contain rare-earth lanthanides, offer a promising alternative. These particles convert near-infrared light into visible emissions and stand out for their strong emission efficiency, chemical and thermal stability, low toxicity, biological compatibility, and ease of surface modification [21,22]. Unlike traditional fluorescent particles, UC nanoparticles do not self-fluoresce and are less prone to quenching, especially when used in core/shell structures or doped with high concentrations of lanthanides [23,24,25]. As a result, they are useful in diverse applications like drug delivery, photodynamic therapy, biosensing, bioimaging, and optogenetics [21,26,27]. Detecting and mapping plastic NMPs in soil micro-organisms presents additional challenges. Most microscopic imaging methods are limited to small regions at the nano-to-microscale [28]. Prior research has relied on fluorescent labeling, which suffers from high detection thresholds and false positives due to natural fluorescence from tissues of microorganisms. Additionally, NMPs are difficult to identify due to their small size and organ-like appearance. Therefore, new and more reliable detection methods are needed to accurately track plastic NMPs in soil organisms [20].

Recent techniques, like dark-field hyperspectral microscopy (DF-HSI), enable label-free microplastic detection in C. elegans [29] and its reliance on light scattering limits sensitivity (<200 nm) and require unrealistic concentrations (>0.1% *w*/*v*). We overcome these constraints using luminous upconverted nanoparticles that employ near-infrared-to-visible (980 nm → 540/660 nm) anti-Stokes conversion to (1) eliminate autofluorescence, (2) detect nanoparticles at environmental doses (0.1–100 μg/L), and (3) dose-dependent biodistribution, enabling the first nanoplastics tracking at biologically relevant concentrations.

In this study, lanthanide-doped upconversion nanoparticles (NaYF^4^:Yb^3+^/Er^3+^) were synthesized to visualize, identify, and compare the ingestion behavior of nanoplastics and microplastics in soil-dwelling worms. Upconversion-labeled luminous polystyrene nanoparticles (PS@LUC-nano) and microparticles (PS@LUC-micro) were utilized to track and compare the ingestion of NMPs in *C. elegans*. Additionally, the nematodes were exposed to pristine polystyrene NPs and MPs at varying concentrations to assess and compare physiological effects, including locomotion, reproduction, and body length. The synthesis and application of NaYF_4_:Yb^3^⁺/Er^3^⁺ as a fluorescent probe offers a novel approach for visualizing the ingestion of NPs and MPs in biological systems. In addition, this study simultaneously investigates the ingestion and physiological impacts of both NPs and MPs, which allows for a direct comparative assessment of size-dependent effects in *C. elegans*. Employing PS@LUC-nano and PS@LUC-micro in a soil nematode represents a novel application for environmental exposure assessment, particularly in the context of soil ecosystems, which are less frequently studied than aquatic systems. Moreover, the integration of real-time fluorescence imaging with toxicological testing bridges a critical gap between exposure visualization and functional outcomes, providing a powerful method to track and understand particle-organism interactions.

## 2. Materials and Methods

### 2.1. Materials

Yttrium trichloride hexahydrate (YCl_3_.6H_2_O, 99%), Erbium trichloride hexahydrate (ErCl_3_.6H_2_O, 99%), ytterbium trichloride hexahydrate (YbCl_3_.6H_2_O, 99%), sodium hydroxide (NaOH, 98%), ammonium fluoride (NH_4_F, 99.99%), cyclohexane (C_6_H_12,_ 99.5%), Ethanol (ET, 99.5%), Acetone, and Oleic acid (OA) (technical grade, 90%) were purchased from Sigma-Aldrich (Shanghai, China). Polystyrene nanoparticles (PS-NPs) and microparticles (PS-MPs) suspension in ultrapure water with a concentration of 25 mg/mL, with the diameter of 50 nm and 1 µm were obtained from Yuan Biotech (Shanghai, China). The *Caenorhabditis elegans* (*C. elegans*) strain used in the study was the wild type (Bristol strain N2). Both the *C. elegans* strain and its bacterial food source, *Escherichia coli* (*E. coli*) OP50, were sourced from the Caenorhabditis Genetics Center in Minneapolis, MN, USA.

### 2.2. Fabrication of Luminous Polystyrene Upconverted NPs and MPs

Lanthanide doped luminous polystyrene upconverted nanoparticles and microparticles (PS@LUC-nano, PS@LUC-micro) was fabricated by procedure mentioned in our previous studies [21,22]. Briefly for PS@LUC-micro, 3 mg NaYF_4_:Yb^3+^/Er^3+^ (LUC-nano) (detailed procedure mentioned in Text S1) was dispersed in methanol (3 mL) by sonication of 15 min and labeled as solution one. To prepare the second solution, a suspension of pristine PS particles (1 µm) was sonicated for 10 min to prevent aggregation. Next, 0.8 mL of this suspension, containing 20 mg of PS-MPs, was mixed with 9.2 mL of acetone (acting as a swelling agent), and the pH was adjusted to 7.0 using 2 M NaOH. The first solution was then heated to 90 °C under magnetic stirring before being added to the second solution. After stirring for 40 min, the mixture was allowed to cool gradually to room temperature for deswelling. Finally, the solution was filtered through a borosilicate glass fiber filter to yield luminescent PS microparticles. To ensure that no excess LUC nanoparticles were present to contribute to the overall luminescence, the filtration process was performed three times. After each filtration, the filtrate was carefully discarded. The final filtrate was collected and analyzed for luminescence via spectrophotometer, with no significant luminescence detected after the third filtration cycle, thereby confirming the removal of excess LUC nanoparticles (Figure S1). This repeated filtration process guarantees that the observed luminescence originates exclusively from the PS@LUC-nano particles, rather than from any free, unbound LUC nanoparticles. The same procedure was followed to produce PS@LUC-nano. The characterization methods of fabricated materials are mentioned in Text S2.

### 2.3. Cultivation and Synchronization of Worm C. elegans

The *C. elegans* cultures were maintained on nematode growth medium (NGM), composed of 3 g/L NaCl, 2.5 g/L peptone, 17 g/L agar, 25 mL/L of 1 M potassium phosphate, 1 mL/L of 1 M CaCl_2_·2H_2_O, 1 mL/L of 1 M MgSO_4_·7H_2_O, and 1 mL/L cholesterol. *E. coli* OP50 was used as the food source. The worms were grown and propagated at 22 ± 2 °C. To transfer worms to fresh plates, the chunking method was used: a sterilized spatula was used to move a piece of the old agar containing worms to a new NGM plate with an OP50 lawn. Transfers were performed every 3–4 days to prevent contamination and maintain healthy growth conditions.

Synchronization of *C. elegans* was performed using the bleaching method. Gravid adults were washed from NGM plates using M9 buffer and collected in a 2 mL centrifuge tube. The worms were allowed to settle by gravity, after which the supernatant was removed. Then, 1.5 mL of nematode lysis solution was added (prepared from 10 mL of 2× worm lysis buffer: 5 mL ddH_2_O, 1 mL 10 M NaOH, and 4 mL Clorox bleach). The tube was gently vortexed for around 5 min using a shaker. The sample was then centrifuged at 3000 rpm for 1 min, and the supernatant was discarded. M9 buffer was used to wash the pellet, and this washing step was repeated twice to remove any remaining lysis solution. After discarding the final supernatant, the egg pellet was gently resuspended and transferred onto a fresh NGM plate seeded with OP50. The plate was then incubated at 22 ± 2 °C.

### 2.4. Luminous Upconverted PS Particles Exposure with C. elegans

In this study, luminous upconverted polystyrene particles PS@LUC-nano (PS@NaYF_4_:Yb^3+^/Er^3+^; fabricated using PS-NPs sized 50 nm) and PS@LUC-micro (PS@NaYF_4_:Yb^3+^/Er^3+^; fabricated using PS-MPs sized 1 μm) were used to visualize the ingestion behavior of nanoplastics and microplastics in *C. elegans*. The exposure concentrations of PS@LUC-nano and PS@LUC-micro were 0.1 μg/L, 1 μg/L, 10 μg/L and 100 μg/L. The exposure experiments were carried out in 35 mm diameter disposable culture dishes, each containing 1 mL of NGM culture medium. To prepare the plates, 50 μL of OP50 bacterial media was mixed with the required concentration of labeled PS (PS@LUC-nano or PS@LUC-micro) and then applied to the NGM agar plates, which were left to dry at room temperature for worm culture. Afterward, the synchronized *C. elegans* eggs were placed on the prepared culture plates and incubated at 22 ± 2 °C for 42 h. Each treatment group had three replicates (*n* = 3). The blank control group was treated with an equivalent amount of K solution (3.1 g/L NaCl; 2.384 g/L KCl).

### 2.5. Visualization of PS@LUC-Nano and PS@LUC-Micro in C. elegans

Wild-type *Caenorhabditis elegans* at the L4 developmental stage were harvested from culture dishes containing the nanoparticle exposure solution. The worms were dislodged and subsequently rinsed three times with sterile M9 buffer to remove any surface-adhered particles. Cleaned specimens were mounted on standard glass microscope slides with a minimal volume of buffer and covered with coverslips.

Luminescence imaging was conducted using a Leica S9D stereo microscope, equipped with a Canon EOS RP digital camera. A 20× long-working-distance objective lens (numerical aperture 0.45) was used for optimal visualization of internalized particles. The standard illumination system was replaced with a 980 nm near-infrared (NIR) diode laser (adjusted to ~1 W/cm^2^ at the sample plane) to excite upconversion nanoparticles. A green bandpass emission filter (400–550 nm) was fitted to the optical path to selectively detect upconverted luminescence.

Images were captured with the Canon EOS RP in manual mode. The exposure time was set between 5 and 15 s, depending on luminescence intensity, with ISO 800 [30] and aperture f/5.6. All samples were imaged under identical settings to ensure comparability. Image acquisition and basic processing were conducted using MP97 (Mp97-03) software. For post-acquisition analysis and overlay of luminescence with brightfield images, ImageJ software (1.54 g, Java 1.8.0_345 64 bit) was used.

### 2.6. Effects of PS-NPs and PS-MPs Exposure on the Physiology of C. elegans

#### 2.6.1. Assessment of the Worm Body Length

The synchronized worm eggs were transferred to culture dishes containing exposure solutions of PS-NPs (50 nm) and PS-MPs (1 μm) with concentrations of 0 μg/L, 0.1 μg/L, 1 μg/L, 10 μg/L and 100 μg/L, respectively. The cultures were cultivated to L4 stage at 22 ± 2 °C for 42 h. Using a worm picker, we randomly selected 30 worms in each group at a designated exposure time. The selected worms were transferred to a 5 mL centrifuge tube and subjected to a water bath at 55 ± 1 °C for 5 min. When the worms became stiff, they were transferred from the tube bottom to a glass slide by using a 10 μL pipette. The worms were then imaged by placing a coverslip over the slide and observing the sample under a stereomicroscope (Mshot MZX-100, Guangzhou Micro-shot Technology Co., Ltd., Shanghai, China). The ImageJ software (1.54 g, Java 1.8.0_345 64 bit) was used to measure the length of each worm.

#### 2.6.2. Assessment of the Worm Brood Number

The synchronized worm eggs were transferred to culture dishes containing exposure solutions of PS-NPs (50 nm) and PS-MPs (1 μm) with concentrations of 0 μg/L, 0.1 μg/L, 1 μg/L, 10 μg/L and 100 μg/L, respectively. The cultures were cultivated to L4 stage at 22 ± 2 °C for 42 h. Using a worm picker, we transferred L4 stage worms to new NGM plates with exposure solution. Each treatment group had six culture plates. At 24 h intervals, the worms were transferred to fresh NGM plates. The original plates were continuously cultured for 48 h to allow the eggs to hatch and develop to the L4 stage. The number of offspring worms at this stage was visualized and recorded by the Mshot MZX-100 stereomicroscope (Guangzhou Micro-shot Technology Co., Ltd., Shanghai, China). The steps were repeated until the worms stopped laying eggs. The offspring numbers from all plates were summarized to obtain the total brood size of the worms.

#### 2.6.3. Assessment of Worm Locomotion Behavior

The synchronized worm eggs were transferred to culture dishes containing exposure solutions of PS-NPs (50 nm) and PS-MPs (1 μm) with concentrations of 0 μg/L, 0.1 μg/L, 1 μg/L, 10 μg/L, and 100 μg/L, respectively. The cultures were grown at 22 ± 2 °C for 42 h until the worms reached the L4 stage. Using a worm pick, the L4-stage worms were transferred to a clean NGM culture dish without an OP50 lawn. After a 1 min recovery period, the worms were observed under a dissecting microscope. A 25 s video of their movements was recorded using the MZX-100 stereomicroscope. The worms’ movement trajectories, head thrashes, and body bending frequencies were analyzed using WormLab 2024 software (MBF Bioscience, Williston, VT, USA).

### 2.7. Statistical Analyses

The data were analyzed using two-way analysis of variance (ANOVA). Statistical analysis was conducted with IBM SPSS Statistics 2022 (v27.0.1) and Microsoft Excel 365. Differences were considered statistically significant when *p* < 0.05 or *p* < 0.01. The results are presented as mean ± standard deviation (s.d.). The figures were generated using OriginPro 2021.

## 3. Results and Discussion

### 3.1. Fabrication and Characterization of Luminous Polystyrene Upconverted Particles

**Figure 1:**
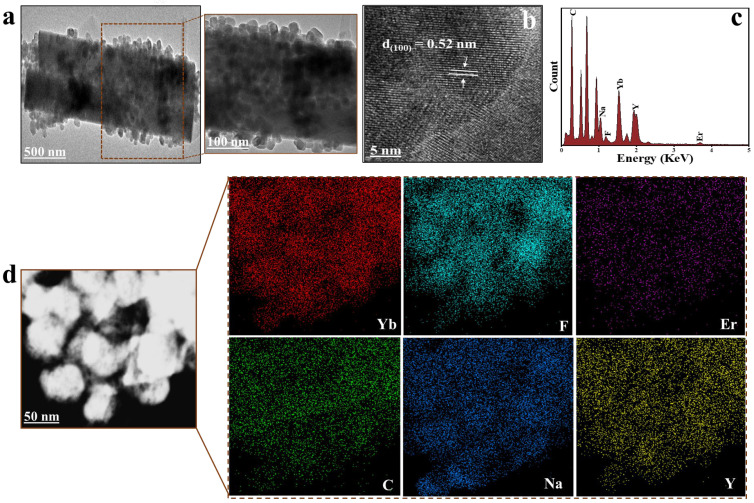
Structure and morphology of PS@LUC-micro. (**a**) TEM image. (**b**) d-spacing of PS@LUC-micro. (**c**) EDX elemental graph. (**d**) TEM EDX elemental mapping.

The morphology and structure of LUC-nano, PS@LUC-nano, and PS@LUC-micro were analyzed using TEM and FT-IR. TEM images of the synthesized LUC-nano (Figure S2) revealed that these particles were cubic in shape and uniformly dispersed, which is consistent with previous investigations [31,32,33]. The elemental intensity graph and corresponding elemental maps for Na, Y, F, Yb, and Er confirmed the homogeneous doping of Yb^3+^ and Er^3+^ into the NaYF_4_ host lattice. The TEM images, elemental intensity graphs, and elemental maps for Na, Y, F, Yb, Er, and C clearly illustrated that LUC-nano were uniformly distributed on the surface of PS-NPs (Figure S3) and PS-MPs (Figure 1). Additionally, the TEM-EDX analysis confirmed the successful incorporation of LUC nanoparticles into both PS@LUC-nano and PS@LUC-micro particles. The quantified weight% of the LUC nanoparticles to the PS matrix in Table S1 indicate significant incorporation efficiency. Moreover, Figure 1b and Figure S3b exhibit clear lattice fringes with a 0.52 nm spacing, consistent with the cubic NaYF_4_ planes [23,34]. The hydrodynamic diameters of the synthesized PS@LUC-nano and PS@LUC-micro were measured using dynamic light scattering (DLS), yielding values of 208 ± 6 nm and 3.6 ± 0.7 μm, respectively (Figure S4). The zeta potential of the pristine PS (nano), PS (micro), PS@LUC-nano, and PS@LUC-micro particles were found to be −34.7 mV, −32.5 mV, −22.6 mV, and −19.9 mV, respectively.

**Figure 2:**
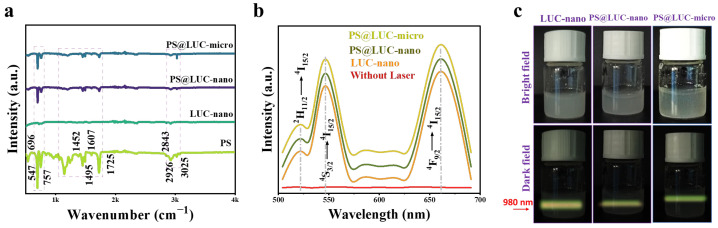
Structural and optical characterization of PS@LUC-micro and PS@LUC-nano. (**a**) FTIR spectra. (**b**) The luminescent upconversion emission spectra of samples. (**c**) Digital photos of PS@LUC-nano and PS@LUC-micro captured in bright field and dark field under 980 nm laser excitation.

Figure 2a presents the FTIR analysis, showcasing the spectra for LUC-nano, PS, PS@LUC-nano, and PS@LUC-micro. The PS spectrum reveals distinct peaks, indicating the presence of specific functional groups, with clearly defined vibrational energies that establish a baseline for understanding structural changes upon incorporation. The FTIR spectrum of LUC-nano, before the inclusion of PS, highlights peaks corresponding to the vibrational modes of various bonds within the α-NaYF_4_:Yb^3+^/Er^3+^ matrix [25,35,36]. These characteristic peaks provide a reference point for assessing any structural alterations. Notably, the significant reduction or enhancement of specific peaks in the PS@LUC-nano and PS@LUC-micro spectra suggests interactions between the luminous upconverted nanoparticles and the incorporated PS-NPs or PS-MPs. These changes likely reflect modifications in the surface chemistry of the LUC-nano upon nanoparticle inclusion.

The optical properties of α-NaYF_4_:Yb^3+^/Er^3+^ are primarily governed by its cubic crystal structure. The symmetry and stability of the cubic lattice facilitate efficient energy transfer between Yb^3+^ and Er^3+^ ions, enhancing upconversion efficiency and photoluminescence stability. This makes the material highly suitable for bioimaging applications [20,24,37]. α-NaYF_4_:Yb^3+^/Er^3+^ is regarded as one of the most effective materials for converting near-infrared (NIR) light into visible light [26,38]. Figure 2b illustrates the upconversion (UC) luminescence emission spectra of LUC-nano, PS@LUC-nano, and PS@LUC-micro, measured at an excitation wavelength of 980 nm. This excitation is absorbed by Yb^3^⁺ ions, which function as sensitizers. The Yb^3^⁺ ions possess a straightforward energy level structure with a ground state and one excited state (^2^F_7/2_ and ^2^F_5/2_), enabling efficient absorption of NIR light [38]. Upon excitation, energy is transferred from Yb^3^⁺ ions to Er^3^⁺ ions, which possess multiple excited states, resulting in a broad and rich emission spectrum in the visible range. Key emission peaks for Er^3^⁺ in α-NaYF_4_:Yb^3+^/Er^3+^ typically appear around 525 nm, 540 nm (green region), and 660 nm (red region). These emissions correspond to transitions from higher excited states to lower energy levels of Er^3^⁺ [25,39]. Specifically, the emissions at 540 nm and 660 nm are attributed to the transitions ^4^S_3/2_ → ^4^I_15/2_ and ^4^F_9/2_ → ^4^I_15/2_, respectively. Notably, the UC photoluminescence intensity of α-NaYF_4_:Yb^3+^/Er^3+^ with PS-NPs and PS-MPs remains unquenched, indicating that the energy transfer is highly efficient. This is due to the excellent spectral overlap between the emission of Yb^3^⁺ and the absorption of Er^3^⁺ [25,40]. Figure 2c presents dark-field upconverted photoluminescence images of LUC-nano, PS@LUC-nano, and PS@LUC-micro, excited by 980 nm NIR light. The nanoparticles emit a yellowish-green light, which is a mixture of both green and red emissions. The green and red emissions arise from the transitions ^2^H_11/2_ → ^4^I_15/2_ and ^4^S_3/2_ → ^4^I_15/2_ (green region) and ^4^F_9/2_ → ^4^I_15/2_ (red region) in Er^3^⁺ [27].

### 3.2. Microscopic Visualization of C. elegans

Stereomicroscopes are ideal for bio-imaging applications due to their non-invasive nature, enabling real-time observation of upconverted nanoparticles without causing significant photodamage. By utilizing appropriate optical filters, the visible emissions from the upconverted nanoparticles, stimulated by the 980 nm NIR light, can be clearly identified, minimizing interference from other light sources [41]. The labeling of PS-NPs and PS-MPs with luminescent upconverted nanoparticles is expected to enhance the study of plastic particle ingestion by *C. elegans* when compared to traditional fluorescence methods. Key attributes such as enhanced photostability [42], superior signal-to-noise ratio [43], and low toxicity [44] allow for more accurate, reliable, and complex investigations into the interactions between NMPs and biological entities.

**Figure 3:**
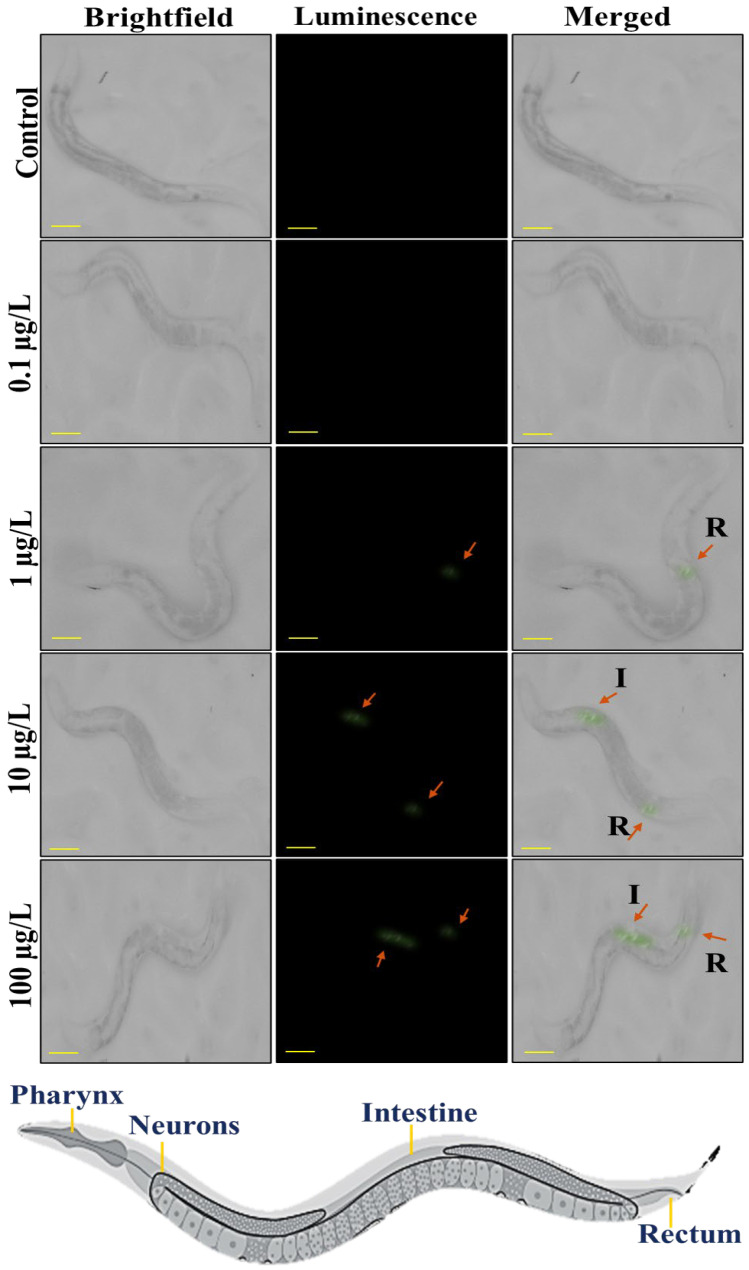
Stereomicroscopic visualization of ingestion of PS@LUC-micro under 980 nm NIR illumination. Bright field, dark field, and merged images of *C. elegans* incubated with 0 μg/L, 0.1 μg/L, 1 μg/L, 10 μg/L, and 100 μg/L of PS@LUC-micro (I: Intestine, R: Rectum). The scale bar is 100 µm.

The conventional techniques suffer from limited sensitivity for nanoplastics due to weak light scattering [29], the presented upconverted luminescence probes overcome this fundamental limitation through near-infrared-to-visible (NIR-vis) photon upconversion. This innovative approach provides three key advantages of complete elimination of autofluorescence interference through anti-Stokes emission and visualization at environmentally relevant concentrations (0.1–100 μg/L). The synthesized luminous PS@LUC-nano and PS@LUC-micro nanoparticles were utilized to enhance their detectability by effectively eliminating autofluorescence from the cells of *C. elegans* when excited by a 980 nm NIR laser source. This approach enabled the capture of highly specific images with minimal background noise, ensuring clearer visualization of the nanoparticles [39,45,46]. The 980 nm NIR excitation emits low-energy photons, which are converted into high-energy visible light emissions, significantly reducing the interference from background fluorescence in the organism’s body cells. To confirm that the observed luminescence was solely due to the PS@LUC nanoparticles, a blank control (*C. elegans* with no particles) was included in all experiments. This control was subjected to the same 980 nm irradiation to ensure no luminescence arose from *C. elegans* or the irradiation itself. As depicted in Figure 3, no luminescence from PS@LUC-micro was observed in worms at doses of 0 μg/L and 0.1 μg/L. A faint green signal appeared at a dose of 1 μg/L. In contrast, at higher doses of 10 μg/L and 100 μg/L, the nanoparticles emitted significantly more intense luminescence, particularly in the posterior regions of the worm intestine. This suggests that the microparticles are accumulating in the intestinal lumen, where digestion and absorption processes take place [16,44,47].

**Figure 4:**
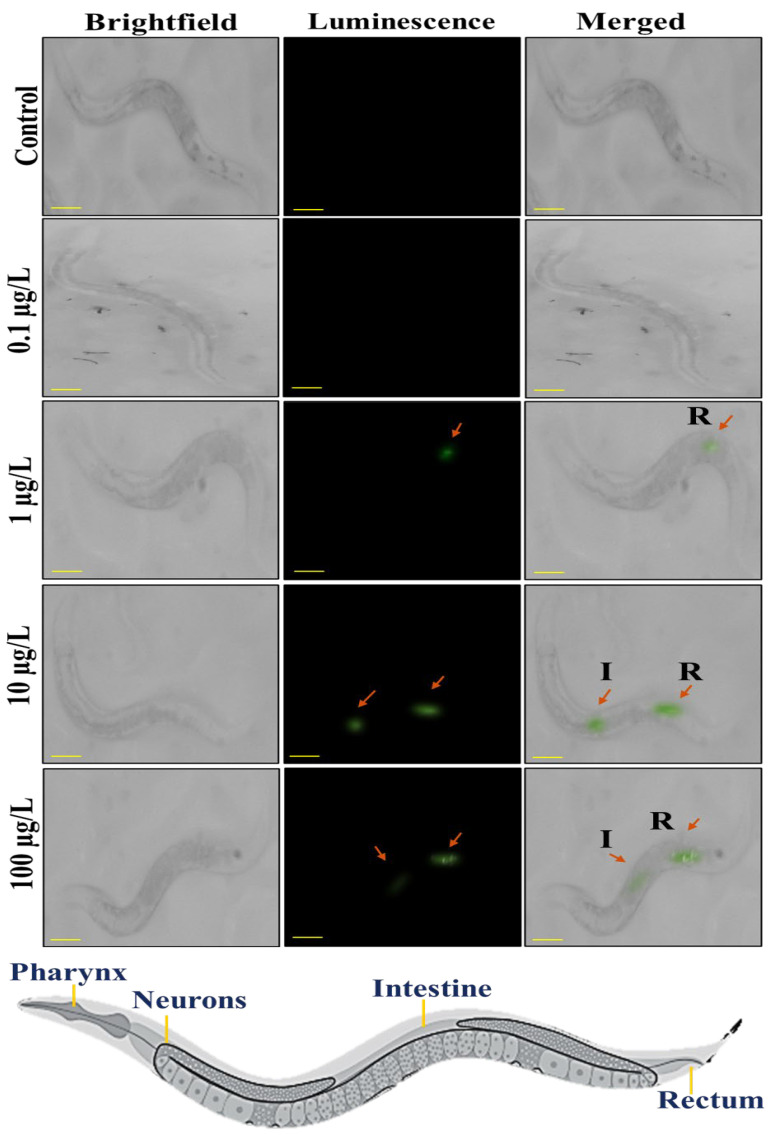
Stereomicroscopic visualization of ingestion of PS@LUC-nano under 980 nm NIR-illumination. Bright field, dark field, and merged images of *C. elegans* incubated with 0 μg/L, 0.1 μg/L, 1 μg/L, 10 μg/L, and 100 μg/L of PS@LUC-nano (I: Intestine, R: Rectum). The scale bar is 100 µm.

Figure 4 illustrates the microscopic images of *C. elegans* after exposure with different concentrations of PS@LUC-nano. As it is shown that at doses of 0 μg/L and 0.1 μg/L luminescence signal was not observed in worms. PS@LUC-nano at doses of 10 μg/L and 100 μg/L shows more intense luminescence in the intestine region and tail region of the worms.

PS@LUC-nano at doses of 10 μg/L and 100 μg/L shows more intense luminescence in the pharynx, intestine region and tail region of the worms. Luminous signals from Figure 3 and Figure 4 show that the gastrointestinal tract of *C. elegans* has great permeability to nanoparticles compared to microparticles, hence enhancing their absorption. Moreover, nanoparticles may engage with the intestinal microvilli or enterocyte cells of the digestive epithelium, facilitating their internalization [48]. Upon ingestion, nanoparticles may permeate the intestinal walls and then enter the hemolymph, circulating throughout the organism [16,47]. The posterior area may exhibit reduced fluorescence relative to the intestines. The detection of luminescence in the front and posterior regions may suggest that the particles are circulating inside the body, maybe migrating from the digestive system to other areas of worm. The posterior area may exhibit reduced fluorescence relative to the intestines [47,49].

### 3.3. Physiological Impacts of PS-NPs and PS-MPs Exposure in C. elegans

#### 3.3.1. PS-NPs and PS-MPs Impact on Locomotion Behavior of *C. elegans*

**Figure 5:**
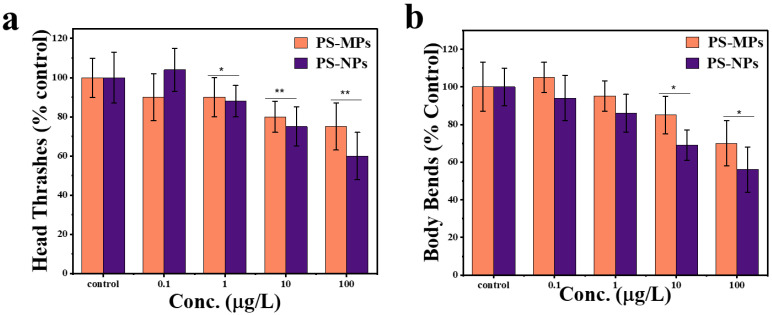
Physiological effects on locomotion behavior of worms following exposure to PS-MPs and PS-NPs at varying concentrations. (**a**) Effect of PS-MPs and PS-NPs on head thrashes (% control) of *C. elegans* after exposure. (**b**) Effect of PS-MPs and PS-NPs on body bends (% control) of *C. elegans* after exposure. The bars represent mean ± standard error of the mean (SEM), *n* = 30 per group. * *p* < 0.05 and ** *p* < 0.01 indicate significant differences between PS-MPs and PS-NPs.

The physiological effects of PS-NPs and PS-MPs exposure at different concentrations on body bends and head thrashes were investigated in *C. elegans*. As illustrated in Figure 5, subacute exposure to 0.1–1 μg/L of PS-MPs and PS-NPs did not obviously affect body bends and head thrashes. The analysis of *C. elegans* exposed to PS-NPs and PS-MPs demonstrates a distinct dose-dependent reduction in locomotor behaviors such as head thrashes (Figure 5a) and body bends (Figure 5b).

Exposure to PS-NPs resulted in a more pronounced decrease in head thrash frequency compared to PS-MPs at concentrations of 1 µg/L, 10 µg/L, and 100 µg/L, with statistical significance (* *p* < 0.05). This finding supports the conclusion that nanoplastics exhibit higher neurotoxicity affecting locomotor behavior than microplastics. No significant difference was observed at 0.1 µg/L, suggesting that particle size effects become more apparent at higher concentrations (Figure 5a). Exposure to PS-NPs led to a greater reduction in body bends at concentrations of 1 µg/L and 100 µg/L, with statistical significance (* *p* < 0.05). This suggests that PS-NPs are more impactful than PS-MPs in reducing body movement at these concentrations. At concentrations of 0.1 µg/L, no significant differences were observed between PS-MPs and PS-NPs. These findings highlight that nanoplastics may have a more potent effect on physical activity at higher concentrations, indicating concentration-dependent effects of particle size on behavior (Figure 5b).

The measurement of locomotor impairments showed a 40% (PS-NPs) and 25% (PS-MPs) reduction in head thrashes, as compared to control group of worms at the dose of 100 µg/L of PS-NPs and PS-MPs, respectively. Furthermore, a 44% (PS-NPs) and 29% (PS-MPs) decrease in body bends as compared to the control group, suggesting neuromuscular system interactions. As the concentration of PS-NPs and PS-MPs increases, both movement metrics decline, indicating neuromuscular dysfunctional behavior [50]. Nonetheless, PS-NPs have a much larger impact than PS-MPs, indicating that nanoplastics infiltrate biological membranes with enhanced efficacy, resulting in increased bioavailability and toxicity [51]. The decrease in head thrashes and body bends with PS-NP exposure is more significant, possibly attributable to their nanoscale dimensions, which provide enhanced contact with neurons and muscles [50]. Conversely, PS-MPs, because to their greater size, have a comparatively less bioactive effect, but they still induce a reduction in locomotion at elevated doses [52].

**Figure 6:**
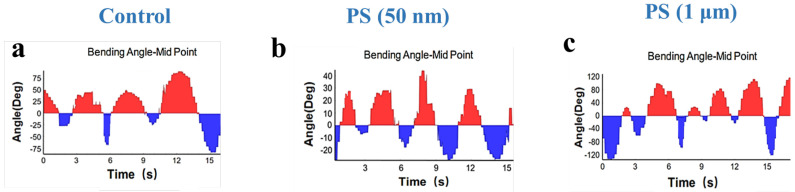
Physiological effects of nanoplastics and microplastics on *C. elegans*. (**a**) The body-bending angle curves of *C. elegans* in control conditions. (**b**) The body-bending angle curves of *C. elegans* after exposure with 100 μg/L of PS-NPs. (**c**) The body-bending angle curves of *C. elegans* after exposure with 100 μg/L of PS-MPs.

Figure 6 depicts the effect of PS-NPs and PS-MPs on the locomotion of *C. elegans* by examining the bending angle-midpoint with time. The bending angle analysis revealed distinct locomotor patterns across groups. The control group (Figure 6a) demonstrates a stable oscillation pattern with a uniform range of motion. It indicates normal neuromuscular activity with a consistent bending amplitude of 45° ± 3° and low Erratic Movement Index (EMI) values (0.2 ± 0.1), reflecting coordinated neuromuscular activity. In contrast, worms exposed to PS-NPs (Figure 6b) exhibit a significant decrease in bending angles, characterized by diminished amplitude (25° ± 5°) and erratic movement patterns alongside elevated EMI (0.7 ± 0.2), characterized by irregular pauses (“freezing”) and disrupted body waves, suggesting impaired neural signals. It indicates compromised neuromuscular coordination, presumably attributable to the capacity of nanoplastics to infiltrate tissues and alter neuronal communication [53]. PS-MPs (Figure 6c) exposure leads to a milder decline in bending angles with amplitudes of 35° ± 4° and mildly elevated EMI (0.3 ± 0.1), suggesting that microplastics largely induce mechanical interference rather than direct neural damage [52,54]. The size-dependent effects revealed underscore the enhanced bioavailability and cellular contact of nanoplastics, rendering them more perilous than microplastics. Although PS-MPs are mostly restricted to the gastrointestinal tract, PS-NPs may permeate biological tissues, resulting in oxidative stress, mitochondrial malfunction, and increased locomotor impairment [55]. These results underscore the increased ecological hazards associated with nanoplastics, since their diminutive size facilitates profound biological interactions and systemic toxicity, prompting worries over their persistent impacts on species and ecosystems [16,52].

#### 3.3.2. PS-NPs and PS-MPs Impact on Reproduction and Growth of *C. elegans*

**Figure 7:**
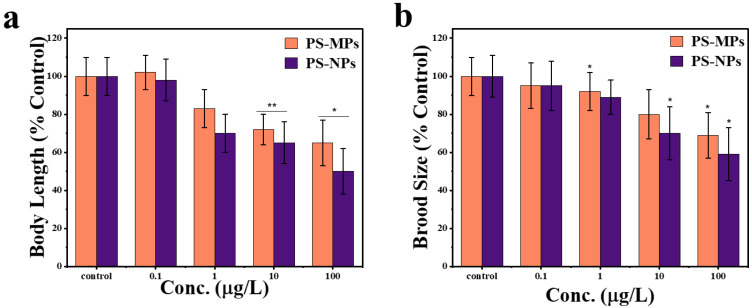
Physiological effects on growth of worms following exposure to PS-MPs and PS-NPs at varying concentrations. (**a**) Effect of PS-MPs and PS-NPs on body length (% control) of C. elegans after exposure. (**b**) Effect of PS-MPs and PS-NPs on brood size (% control) of C. elegans after exposure. The bars represent mean ± standard error of the mean (SEM), *n* = 30 per group. * *p* < 0.05 and ** *p* < 0.01 indicate significant differences between PS-MPs and PS-NPs.

The physiological impacts of PS-NPs (50 nm) and PS-MPs (1 μm) exposure at different concentrations on body length and brood size were investigated in *C. elegans*. As illustrated in Figure 7, subacute exposure to PS-MPs and PS-NPs at doses of 1–100 μg/L obviously reduced body length. At low concentrations (0 and 0.1 µg/L), there is no notable effect on body length. However, as concentrations increase to 10 and 100 µg/L, the body length decreases significantly. PS-MPs caused a significant reduction at 10 µg/L (** *p* < 0.01), while PS-NPs showed a significant effect at 100 µg/L (* *p* < 0.05). This suggests that higher concentrations of both micro- and nanoplastics negatively impact organism growth, with nanoplastics potentially exerting greater toxicity at the highest concentration tested (Figure 7a). The subacute exposure to PS-NPs and PS-MPs at exposure dosages of 0.01 μg/L, 1 μg/L, and 10 μg/L did not show any noticeable disorder in number of eggs, exposure to PS-NPs resulted in a more pronounced decrease in brood size compared to PS-MPs at concentrations of 1, 10, and 100 µg/L, with statistical significance (* *p* < 0.05). This suggests that nanoplastics exert a stronger negative effect on reproductive output than microplastics. At lower concentration (0.1 µg/L), the difference between particle types was not significant. These findings indicate that particle size is a critical factor influencing reproductive toxicity in this model. (Figure 7b).

With an increase in both PS-NPs and PS-MPs concentrations, there is a significant reduction in body length and reproductive output, with PS-NPs demonstrating a much more significant effect. The monitoring of physiological disruptions revealed a 49% reduction in body length for PS-NPs and a 34% reduction for PS-MPs compared to the control group. Additionally, there was a 41% decrease in brood size for PS-NPs and a 31% decrease for PS-MPs at a concentration of 100 µg/L, suggesting metabolic disturbances and possible oxidative stress. The more severe decrease in body length in PS-NP’s exposed worms indicates that nanoplastics adversely affect development and metabolism, perhaps owing to their greater bioavailability and capacity to infiltrate biological tissues [56,57]. Likewise, brood size diminishes significantly with PS-NPs exposure, suggesting reproductive toxicity, perhaps due to oxidative stress, endocrine disturbance, or direct harm to germline cells [58,59]. Conversely, PS-MPs also diminish these characteristics, but to a lower degree, presumably owing to their increased size restricting cellular contact and absorption [5,48]. The findings indicate that nanoplastics provide a more significant ecological and biological threat than microplastics, due to their enhanced capacity to penetrate cells, resulting in increased toxicity [5,60]. This underscores the pressing need for more investigation into the mechanisms of plastic-induced toxicity and viable mitigation techniques to tackle the health and environmental hazards linked to nanoplastics pollution.

### 3.4. Proposed Toxicity Mechanism of PS-NPs and PS-MPs

**Figure 8:**
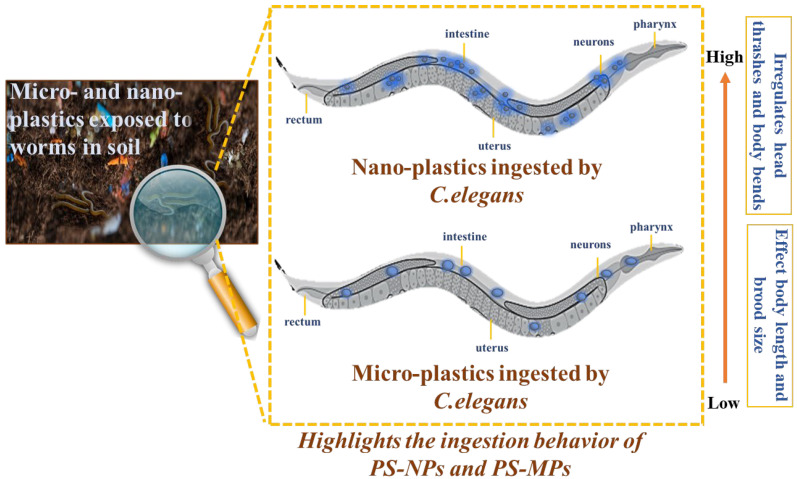
Schematic illustration of micro- and nano-plastic ingestion and physiological impact in *Caenorhabditis elegans*. The magnified panel shows a conceptual exposure scenario of *C. elegans* in a plastic-contaminated soil matrix.

The suggested toxicity mechanisms of PS-MPs and PS-NPs in *C. elegans* (Figure 8) indicate that nanoplastics provide a more significant biological risk than microplastics owing to their reduced size and enhanced bioavailability [55,61]. Upon intake, PS-MPs mostly concentrate in the gastrointestinal tract, leading to mechanical blockage and decreased nutritional absorption, hence indirectly impacting metabolism, development, and reproduction [56,61]. Conversely, PS-NPs may infiltrate intestinal cells, enter systemic circulation, and bioaccumulate in tissues, resulting in oxidative stress, mitochondrial dysfunction, and inflammatory responses [51,62,63]. The impairment of neuromuscular function is shown as erratic head thrashes and body bends, with PS-NPs having a more pronounced impact by disrupting neurotransmission and causing oxidative damage in neurons. Growth suppression is more pronounced with PS-NP exposure, owing to the direct cellular interactions that induce metabolic dysregulation, while PS-MPs primarily hinder growth via starvation. Reproductive toxicity is exacerbated by PS-NP exposure, as these nanoparticles may penetrate germline cells which may significantly reduce brood size [16,58,64]. PS-MPs typically cause localized gut inflammation and nutritional deficiency, while PS-NPs result in systemic toxicity by breaching biological barriers and disrupting many physiological processes [5,65]. These results underscore the heightened ecological danger posed by nanoplastics, necessitating more study on their long-term impacts and the formulation of regulatory measures to alleviate environmental and biological pollution.

## 4. Conclusions

The study successfully utilized luminous upconverted polystyrene nanoparticles (PS@LUC-nano) and microparticles (PS@LUC-micro) to visualize and analyze the ingestion behavior in *C. elegans*. The incorporation method revealed effective visualization of ingested nano- and micro-particles. The photoluminescence stability and cubic lattice symmetry enabled efficient NIR to visible light conversion, making the materials ideal for bio-imaging. Microscopic imaging revealed that micro-particles mostly capture in the digestive tract, but nanoparticles can penetrate intestinal walls, possibly entering systemic circulation and disseminating throughout the worm body. It demonstrated clear distinctions in the impacts of nanoplastics and microplastics, with PS-NPs showing enhanced bioavailability, increased tissue penetration, and elevated systemic toxicity compared to PS-MPs. The investigation observed a dose-dependent decline in locomotor activity, with considerable reductions in head thrashes and body bends in worms subjected to high concentrations (10–100 µg/L) of both PS-NPs and PS-MPs. Locomotor impairments showed a 40% reduction in head thrashes for PS-NPs and 25% for PS-MPs, as well as a 44% decrease in body bends for PS-NPs and 29% for PS-MPs, compared to the control group at 100 µg/L. Physiological disruptions included a 49% reduction in body length for PS-NPs and 34% for PS-MPs, along with a 41% decrease in brood size for PS-NPs and 31% for PS-MPs, suggesting metabolic disturbances and oxidative stress. The results underscore the immediate need for more study on the long-standing impacts of plastic particle exposure on biological systems and the formulation of regulatory policies to address the escalating environmental and health issues linked to nano-plastic pollution. Comprehending these processes will be essential for evaluating the wider ramifications of plastic pollution in ecosystems and its possible threats to human health. Future work will exploit LUC’s tunability to track mixed plastic pollutants and their metabolites, addressing a key gap in environmental nanotoxicology.

## Data Availability

The data presented in this study are available on request from the corresponding author.

## Supplementary Materials

The following supporting information can be downloaded at https://www.mdpi.com/article/10.3390/s25113306/s1. Text S1: Synthesis of lanthanide upconverted nanoparticles; Text S2: Characterization of materials; Figure S1: Luminescence spectra of the PS@LUC-nano and PS@LUC-micro filtrate; Figure S2: Structure and morphology of LUC-nano; Figure S3: Structure and morphology of PS@LUC-nano; Figure S4: Average hydrodynamic diameter of PS@LUC-nano and PS@LUC-micro via DLS; Table S1: TEM-EDX elemental composition (Weight%).
