# Supplementary material for: Luminous Upconverted Nanoparticles as High-Sensitivity Optical Probes for Visualizing Nano- and Microplastics in Caenorhabditis elegans
Source: Sensors (Basel). 2025 May 24;25(11):3306. doi: 10.3390/s25113306 (PMC12157050; doi:10.3390/s25113306)
Supplement: Supplementary file 1 [file sensors-25-03306-s001.zip › sensors-3612019-supplementary.pdf]

Supporting Information

Luminous Upconverted Nanoparticles as High-Sensitivity Optical Probes for Visualizing Nano- and Microplastics in *Caenorhabditis elegans*

Bushra Maryam [1], Yi Wang [1], Xiaoran Li [1], Muhammad Asim [2], Hamna Qayyum [3], Pingping Zhang [4,*] and Xianhua Liu [1,*]

[1] School of Environmental Sciences and Engineering, Tianjin University, Tianjin 300072, China; maryam_bushra@tju.edu.cn (B.M.); wangyi2023@tju.edu.cn (Y.W.); lxr_11281209@tju.edu.cn (X.L.)
[2] Shandong Lead Chemicals Co., Ltd., Linyi 276100, China; asim.muhammad100@yahoo.com
[3] National Synchrotron Radiation Laboratory, University of Science and Technology of China, Hefei 230029, China; hamna141987@mail.ustc.edu.cn
[4] College of Food Sciences and Engineering, Tianjin Agricultural University, Tianjin 300384, China
* Correspondence: zpp@tjau.edu.cn (P.Z.); lxh@tju.edu.cn (X.L.)

Table of Contents

Text S1 Synthesis of lanthanide upconverted nanoparticles.

Text S2 Characterization of materials.

Figure S1 Luminescence spectra of the PS@LUC-nano filtrate and PS@LUC-micro filtrate after three filtration cycles.

Table S1 TEM-EDX elemental composition (Weight%) of LUC-nano, PS@LUC-nano, and PS@LUC-micro

Figure S2 Structure and morphology of LUC-nano. (a) TEM image; (b) d-spacing of LUC-NPs; (c) EDX elemental graph; (d) TEM-EDX elemental mapping.

Figure S3 Structure and morphology of PS@LUC-nano. **(a)** TEM image; **(b)** d-spacing of PS@LUC-nano; **(c)** EDX elemental graph; **(d)** TEM EDX elemental mapping.

Figure S4 Average hydrodynamic diameter of PS@LUC-nano and PS@LUC-micro via DLS. (a) hydrodynamic diameter of PS@LUC-nano and (b) hydrodynamic diameter of PS@LUC-micro.

Text S1 *Synthesis of lanthanide upconverted nanoparticles*

$NaYF_4$:Yb^{3+}/Er^{3+} (18/2 mol%) was prepared based on the previously reported optimized hydrothermal with some modifications [1]. In a typical synthesis process, 1 mL of 0.2 M YCl_3, 0.9 mL of 0.2 M $YbCl_3$, 0.1 mL of 0.2 M $ErCl_3$ stir by magnetic stirrer to prepare homogenous lanthanide (Ln) solution. 0.3 g NaOH (homogenized in 2 mL of ddH_2O), 6 mL of OA, 6 mL of ET, and 2 mL of Ln solution add stepwise in a round bottom flask under magnetic stirring at room temperature (RT). Stir the mixture for 15 min. to obtain the homogenized clear solution. Then, 4 mmol of NH_4F dissolved in 2 mL of ddH_2O was added dropwise to the above mixture to get the milky white solution. After vigorous stirring at RT for 30 min. the whole solution mixture was transferred into a 25 mL Teflon-lined autoclave, sealed tightly, and transferred to pre-heated (200°C) vacuum oven for 10 h. Afterward, the autoclave was allowed to cool under ice-bath. After cooling supernatant was discarded and the final precipitates were obtained after washing with cyclohexane at least three times by the means of centrifugation to remove any possible aggregates of starting materials and dried in vacuum at 60°C to obtain dried $NaYF_4$:Yb^{3+}/Er^{3+} precipitates.

Text S2 *Characterization of materials*

The detailed analyses of the samples were performed using a variety of sophisticated instruments. High-resolution transmission electron microscopy (HR-TEM) images were captured using a JEM-2100F microscope. This microscope was also equipped with an energy dispersive spectrum (EDS) system for elemental analysis. The incorporation of LUC nanoparticles into PS@LUC-nano and PS@LUC-micro particles was analyzed by TEM elemental mapping and elemental intensity graphs. Further, elemental composition was quantified using TEM-EDX elemental composition. The zeta potential and average hydrodynamic diameter of the PS@LUC-micro dispersed in pure water were determined by dynamic light scattering (DLS) using a Malvern Zetasizer Nano-ZS90. Fourier transform infrared spectroscopy (FTIR) measurements were performed in transmission mode at normal incidence, using a Bruker VERTEX 70 spectrometer at ambient conditions. Lastly, the materials' photoluminescence emission spectra were analyzed with the help of F97XP fluorescence spectrophotometer (Shanghai Lengguang, China). This equipment was equipped with a 980 nm NIR laser which serves as the source of excitation.

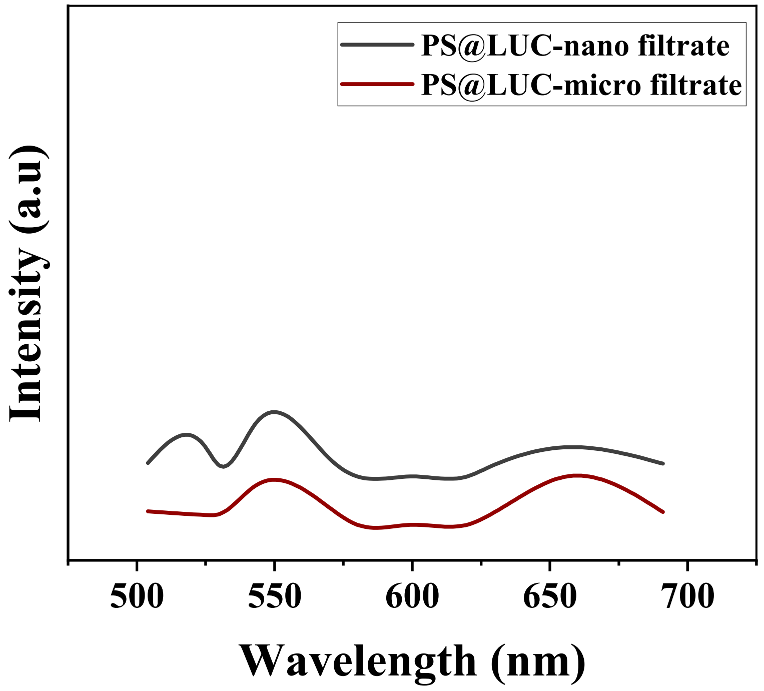

Figure S1 Luminescence spectra of the PS@LUC-nano filtrate and PS@LUC-micro filtrate after three filtration cycles.

Table S1 TEM-EDX elemental composition (Weight%) of LUC-nano, PS@LUC-nano, and PS@LUC-micro

Elements	Weight%		
	**LUC-nano**	**PS@LUC-nano**	**PS@LUC-micro**
F	59.16	45.31	40.22
Na	18.01	17.22	15.12
Y	15.33	11.13	9.91
Yb	7.37	6.11	5.20
Er	0.13	0.11	0.10
C	-	20.12	29.45

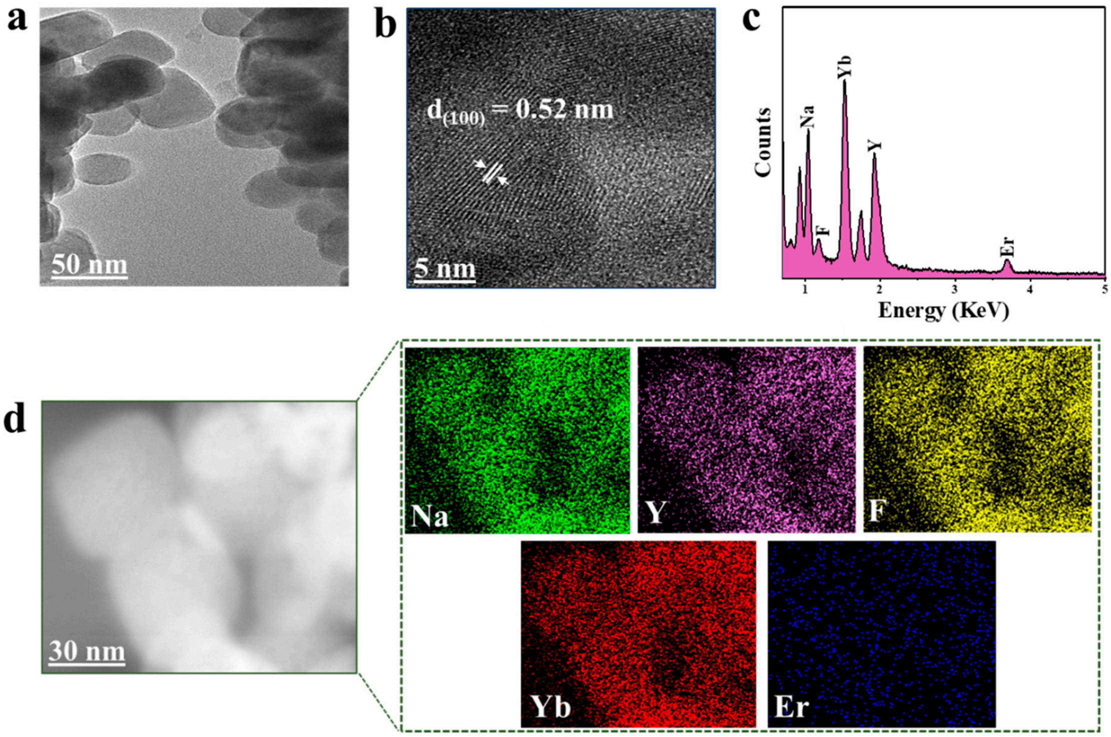

Figure S2 Structure and morphology of LUC-nano. (a) TEM image; (b) d-spacing of LUC-NPs; (c) EDX elemental graph; (d) TEM-EDX elemental mapping.

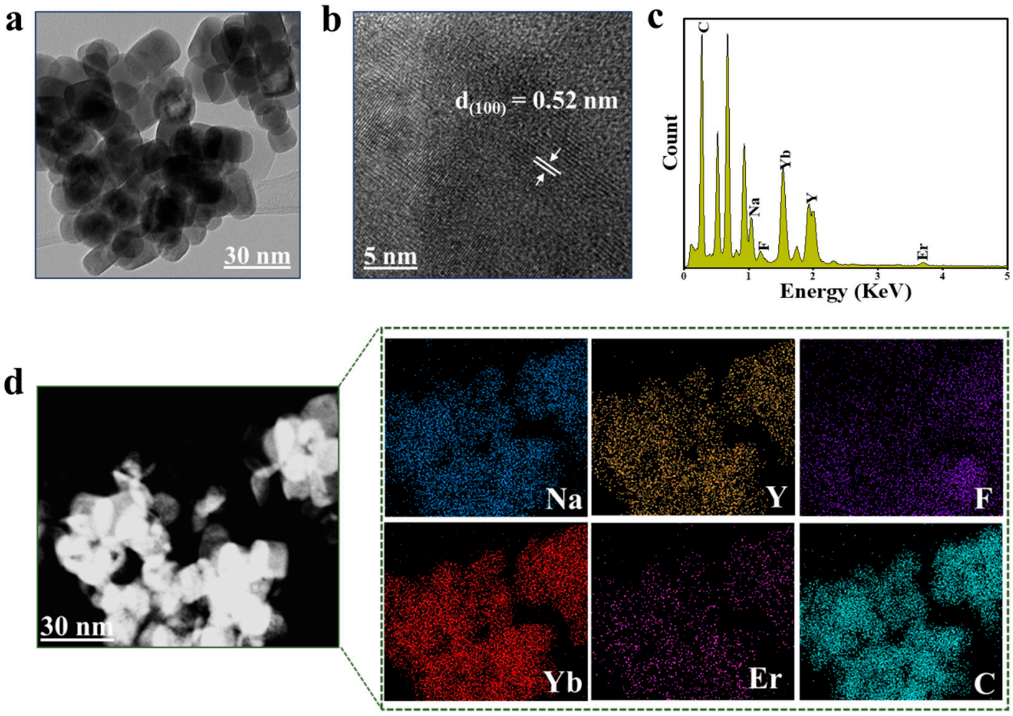

Figure S3 Structure and morphology of PS@LUC-nano. **(a)** TEM image; **(b)** d-spacing of PS@LUC-nano; **(c)** EDX elemental graph; **(d)** TEM EDX elemental mapping.

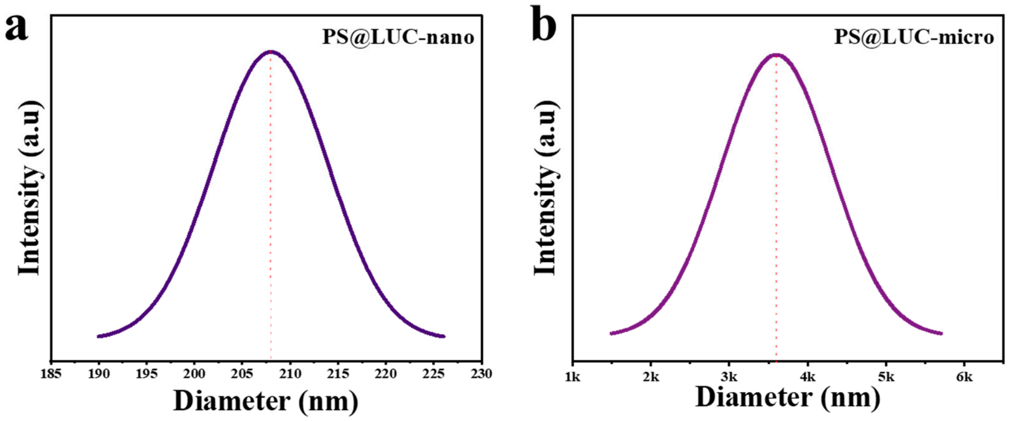

Figure S4 Average hydrodynamic diameter of PS@LUC-nano and PS@LUC-micro via DLS. (a) hydrodynamic diameter of PS@LUC-nano and (b) hydrodynamic diameter of PS@LUC-micro.

Reference

1. Lingeshwar Reddy, K.; Srinivas, V.; Shankar, K.R.; Kumar, S.; Sharma, V.; Kumar, A.; Bahuguna, A.; Bhattacharyya, K.; Krishnan, V. Enhancement of Luminescence Intensity in Red Emitting NaYF4:Yb/Ho/Mn Upconversion Nanophosphors by Variation of Reaction Parameters. *The Journal of Physical Chemistry C* **2017**, *121*, 11783-11793, doi:10.1021/acs.jpcc.7b01334.
